# Characteristics and Prognostic Factors of Venous Thromboembolism in Cancer Patients

**DOI:** 10.3400/avd.oa.22-00036

**Published:** 2022-06-25

**Authors:** Hajime Tsuyuki, Naoto Yamamoto, Naoki Unno, Kazunori Inuzuka, Masaki Sano, Kazuto Katahashi, Tatsuro Yata, Takafumi Kayama, Yuta Yamanaka, Yusuke Endo, Hiroya Takeuchi

**Affiliations:** 1 Division of Vascular Surgery, Hamamatsu Medical Center, Hamamatsu, Shizuoka, Japan; 2 Division of Vascular Surgery, Second Department of Surgery, Hamamatsu University School of Medicine, Hamamatsu, Shizuoka, Japan

**Keywords:** deep vein thrombosis, venous thromboembolism, pulmonary thromboembolism, cancer-associated thrombosis

## Abstract

**Background**: Improving the prognosis of patients with malignant tumors is increasing the number of patients who develop venous thromboembolism. We examined the characteristics and prognostic factors of VTE patients with cancer.

**Methods**: We diagnosed 725 VTE patients from April 2005 to March 2018. There were 322 cancer associated patients (CAT) and 403 non-cancer associated patients (nonCAT). We examined characteristics and prognostic factors of VTE in CAT patients.

**Results**: There were 156 women and 166 men in CAT, and 132 women and 271 men in nonCAT. There was no significant difference in the location of proximal portion of thrombus. When locations were divided into left leg, right leg, and bilateral legs, bilateral cases were more common in CAT group. Comparing the overall survival after VTE diagnosis in the CAT group, the prognosis was poor in patients with high D-dimer level (≧6 µg/mL) along with cancer metastasis and recurrence.

**Conclusions**: Various VTE factors predict prognosis in CAT patients, and CAT is important in the treatment of cancer patients. (This is secondary publication from Jpn J Phlebol 2020; 31(3): 153–159.)

## Introduction

Advances in the treatment of malignant tumors have extended the prognosis of patients. On the other hand, the treatment period for malignant tumors is becoming longer, and the number of patients with venous thromboembolism (VTE) is increasing,^1)^ so the prevention and treatment of VTE is important. Patients with malignant tumors have hypercoagulation, and treatment-related factors, such as surgery, chemotherapy, and central venous catheter placement, are also a risk for VTE.^2)^ They may have different backgrounds compared to VTE in patients with non-malignant tumors. It is well known that patients with malignant tumors with VTE have a poor prognosis,^1)^ but there are few reports on prognostic factors for those patients. Therefore, we investigated patients with malignant tumors with VTE and examined their clinical characteristics and prognostic factors after the onset of VTE. We investigated patient’s concomitant malignant tumor with VTE and examined their clinical characteristics and prognostic factors after the onset of VTE.

## Materials and Methods

The cases included 725 patients diagnosed with VTE from April 2005 to March 2018. There were 322 (44.4%) patients with active malignant tumors (cancer-associated thrombosis; CAT) and 403 patients (55.6%) without malignant tumors (non-cancer-associated thrombosis; nonCAT). The definition of an active malignant tumor follows that proposed by Wells et al.^3)^ We compared age, gender, location of the thrombus, deep vein thrombosis (DVT) symptoms, pulmonary thromboembolism (PTE) symptoms, and reasons for finding VTE between the CAT and nonCAT groups. Furthermore, we examined the prognostic factors of the CAT group for the thrombotic location of DVT, VTE symptoms, D-dimer value, and metastasis/recurrence. Comparisons between the two groups were made by the chi-squared test and Fisher’s exact test, and P<0.05 was considered significant. Risk factors for overall survival were examined by the Kaplan–Meier and Cox proportional hazards models, and P<0.05 was considered significant. We used IBM SPSS Statistics version 26 (IBM, Armonk, NY, USA) as the statistical analysis software.

## Results

The patients’ background is shown in **Table 1**. CAT was observed in patients aged 0.75–93 years, with a mean of 66.1 years. NonCAT was observed in patients aged 10–97 years, with a mean of 64.1 years. There was no significant difference in age at onset between CAT and nonCAT. There was a significant difference between males and females in the CAT group: 166 (51.6%) males and 156 (48.4%) females, compared to 132 (32.8%) males and 271 (67.2%) females in the nonCAT group (P<0.0001). The numbers of proximal DVT were 129 (50.4%) in CAT and 205 (56.5%) in nonCAT. Distal DVT was observed in 127 (49.6%) patients with CAT and 158 (43.5%) patients with nonCAT. There was no significant difference in the location of the thrombus. In CAT, 256 patients had DVT of lower extremities. DVT of the left lower extremity was observed in 100 (39.1%) patients, DVT of the right lower extremity in 97 (37.9%) patients, DVT of the unilateral lower extremity in 197 (77.0%) patients, and DVT of the bilateral lower extremity in 59 (23.0%) patients. In nonCAT, 363 patients had DVT of lower extremities. Left lower extremity DVT was found in 167 (46.0%) patients, right lower extremity DVT in 138 (38.0%) patients, unilateral lower extremity DVT in 305 (84.0%) patients, and bilateral lower extremity DVT in 58 (16.0%) patients. Although patients with nonCAT had more left distal DVT, patients with CAT tended to have more bilateral DVT. When comparing unilateral and bilateral localization, there was significantly more bilateral DVT in CAT (P=0.027). In CAT, 100 (31.1%) patients had symptomatic DVT and 222 (68.9%) patients had asymptomatic DVT. In nonCAT, symptomatic DVT was 158 (39.2%) and asymptomatic DVT was 245 (60.8%). There were significantly more asymptomatic DVT in CAT than in nonCAT (P=0.02).

**Table 1:** Table 1 Patients’ backgrounds

	CAT (n=322)	nonCAT (n=403)	P value
Age (average)	0.75–93 (66.1)	10–97 (64.1)	ns
Gender			
Male	166 (51.6%)	132 (32.8%)	P<0.0001
Female	156 (48.4%)	271 (67.2%)	
Type of DVT			
Lower limb (Proximal)	129 (40.1%)	205 (50.9%)	P=0.13
Lower limb (Distal)	127 (39.4%)	158 (39.2%)	
Upper extremity	23 (7.1%)	17 (4.2%)	
Vena cava	9 (2.8%)	6 (1.5%)	
Splanchnic	4 (1.2%)	1 (0.2%)	
Only PTE	30 (9.3%)	16 (4.0%)	
Distribution of DVT			
Left lower limb	100 (39.1%)	167 (46.0%)	
Right lower limb	97 (37.9%)	138 (38.0%)	
Unilateral lower limb	197 (77.0%)	305 (84.0%)	P=0.027
Bilateral lower limb	59 (23.0%)	58 (16.0%)	
DVT Symptoms			
+	100 (31.1%)	158 (39.2%)	P=0.02
−	222 (68.9%)	245 (60.8%)	
PTE Symptoms			
+	36 (11.2%)	49 (12.2%)	P<0.0001
−	98 (30.4%)	89 (22.1%)	
No image inspection	188 (58.4%)	265 (65.8%)	

DVT: deep vein thrombosis; CAT: cancer associated thrombosis

In CAT, 36 (11.2%) patients had symptomatic PTE, 98 (30.4%) patients had asymptomatic PTE, and 188 (58.4%) patients had no PTE or no examination. In nonCAT, 49 (12.2%) patients had symptomatic PTE, 89 (22.1%) patients had asymptomatic PTE, and 265 (65.8%) patients had no PTE and no examination. As for PTE symptoms, similar to DVT symptoms, asymptomatic PTE was more common in CAT than in nonCAT (P<0.0001). The reasons for the detection of VTE are shown in **Table 2**.

**Table 2:** Table 2 Diagnostic opportunity for VTE

	CAT (n=322)	nonCAT (n=403)	P value
DVT Symptoms	98 (30.4%)	159 (39.5%)	P<0.0001
PTE Symptoms	25 (7.8%)	42 (10.4%)	
Incidental by image	114 (35.4%)	71 (17.6%)	
High D-dimer level	56 (17.4%)	121 (30.0%)	
Screening CUS	29 (9.0%)	10 (2.5%)	

VTE: venous thromboembolism; DVT: deep vein thrombosis; PTE: pulmonary thromboembolism; CUS: compression ultrasonography; CAT: cancer associated thrombosis

In the CAT group, 98 (30.4%) patients had DVT symptoms, 25 (7.8%) patients had PTE symptoms, and 56 (17.4%) patients had high D-dimer levels, whereas in the nonCAT group, 159 (39.5%) patients had DVT symptoms, 42 (10.4%) patients had PTE symptoms, and 121 (30.0%) patients had high D-dimer levels. In the nonCAT group, DVT and PTE symptoms and high D-dimer levels were more frequent reasons for the detection of VTE. On the other hand, incidental detection numbers by imaging were 114 (35.4%) in CAT and 71 (17.6%) in nonCAT, and detection by screening compression ultrasonography numbers were 29 (9.0%) in CAT and 10 (2.5%) in nonCAT; incidental detection of VTE by imaging was more frequent in CAT. **Table 3** summarizes the anticoagulation treatment for VTE. The rate of parenteral anticoagulation was 210 (65.2%) in the CAT group and 244 (60.5%) in the nonCAT group, showing no significant difference. On the other hand, oral anticoagulation was less frequent in CAT, with 188 patients (58.4%) in CAT and 283 patients (70.2%) in nonCAT (P=0.001). This was because some patients did not receive oral anticoagulation as they were in the terminal stage of malignancy, and others received only parenteral anticoagulation as they were unable to ingest orally.

**Table 3:** Table 3 Medicinal treatments for VTE

	CAT (n=322)	nonCAT (n=403)	P value
Parenteral anticoagulants			
+	210 (65.2%)	244 (60.5%)	P=0.196
−	112 (34.8%)	159 (39.5%)	
Oral anticoagulants			
+	188 (58.4%)	283 (70.2%)	P=0.001
−	134 (41.6%)	120 (29.8%)	

VTE: venous thromboembolism; CAT: cancer associated thrombosis

The therapeutic doses of parenteral anticoagulants and oral anticoagulants are shown in **Table 4**. Low-dose parenteral anticoagulation was defined as the subcutaneous injection of unfractionated heparin, low-molecular-weight heparin (LMWH), or Xa inhibitors at doses used for DVT prevention. Low-dose oral anticoagulation was defined as a lower dose of direct oral anticoagulants (DOACs) than recommend in the instructions for use owing to the risk of bleeding, and warfarin with a prothrombin time-international normalized ratio target around 1.5. Regarding parenteral anticoagulants, 62 (29.5%) patients in the CAT group and 38 (15.6%) patients in the nonCAT group were treated with low doses. Low-dose anticoagulation was more common in CAT. In terms of oral anticoagulants, 15 (8.0%) patients in the CAT group and 41 (14.5%) patients in the nonCAT group were treated with low-dose anticoagulants, and low-dose anticoagulation was less common in CAT. For oral anticoagulants, there was no difference in the use of warfarin and DOACs. We usually treat CAT with anticoagulation as aggressively as we do for patients with nonCAT. One of the reasons that parenteral anticoagulation at low doses was often used in CAT is that enoxaparin, which is used for postoperative VTE prophylaxis, was often used in cases of asymptomatic localized thrombus in the leg detected by screening after abdominal pelvic visceral surgery. On the other hand, the reason why low-dose oral anticoagulation was more common in nonCAT is that edoxaban, which is used for postoperative VTE prophylaxis, was often used in cases of asymptomatic localized calf thrombus detected during postoperative orthopedic screening. Patients with CAT who could be treated with oral anticoagulation were often treated with regular-dose anticoagulation. However, some patients treated with parenteral anticoagulation were unable to take food orally, and many of them chose low-dose anticoagulation owing to the risk of bleeding.

**Table 4:** Table 4 Treatment details

	CAT (n=322)	nonCAT (n=403)	P value
Parenteral anticoagulants			
Normal dose	148 (70.5%)	206 (84.4%)	P=0.0004
Low dose	62 (29.5%)	38 (15.6%)	
Oral anticoagulants			
Normal dose	173 (92.0%)	242 (85.5%)	P=0.033
Low dose	15 (8.0%)	41 (14.5%)	
Warfarin	121 (64.4%)	184 (65.0%)	P=0.884
DOAC	67 (35.7%)	99 (35.0%)	

DOAC: direct oral anti coagulants; CAT: cancer associated thrombosis

We examined the patients’ background of CAT. At the time of VTE onset, 110 (34.2%) patients had metastasis or recurrence. The location of onset was outside the hospital in 124 (38.5%) patients. In-hospital cases were 198 (61.5%), of which 70 were non-perioperative cases, 91 were postoperative cases, and 37 were preoperative cases. Chemotherapy was administered to 108 (33.5%) patients at the time of onset. Gastroenterology was the most common tumor area with 144 (44.7%) patients, followed by gynecology with 53 (16.5%) patients, urology with 38 (11.8%) patients, and respiratory medicine with 21 (6.5%) patients (**Table 5**). By tumor organ, the colon was the most common site with 36 patients, followed by the esophagus with 34 patients, the uterus with 29 patients, the rectum with 28 patients, the ovary with 24 patients, the lungs with 21 patients, the stomach with 20 patients, and the prostate with 17 patients.

**Table 5:** Table 5 Backgrounds of CAT patients

Department	n=322	Organ	n=322
Gastroenterology	144 (44.7%)	Colon	36
		Esophagus	34
		Rectum	28
		Stomach	20
		Liver	12
		Biliary tract	8
		Pancreas	8
Gynecology	53 (16.5%)	Uterus	29
		Ovary	24
Urology	38 (11.8%)	prostate	17
		Urinary tract	14
		kidney	6
		Testis	1
Pulmonology	21 (6.5%)	lung	21
Hematology	16 (5.0%)	Hematopoietic organ	16
Otolaryngology	15 (4.7%)	Head and neck	15
Neurology	9 (3.0%)	Brain	9
Mammology	8 (2.5%)	Breast	8
Dermatology	8 (2.5%)	skin	8
Orthopedics	6 (1.9%)	Soft tissue	6
Endocrinology	4 (1.2%)	Endocrine	4

The mean observation period after VTE detection in 322 patients with CAT was 875 days, with a median of 469 days. The mortality risk factors for overall survival were examined in terms of thrombosis factors, metastasis, and recurrence (**Table 6**). Univariate analyses showed that symptomatic DVT was associated with significantly poorer survival (P=0.007, hazard ratio [HR]: 0.650, 95% confidence interval [CI]: 0.477–0.887). In terms of the thrombus location, survival was significantly worse in patients with proximal DVT (P=0.001, HR: 0.596, 95% CI: 0.434–0.818). When the D-dimer level at the time of VTE diagnosis was divided into two groups, those with a D-dimer level of more than 6 µg/mL and those with a D-dimer of less than 6 µg/mL, the former was a significantly poor prognostic factor (P<0.001, HR: 0.493, 95%CI: 0.345–0.704). Among patients with CAT, 110 patients had metastasis or recurrence at the time of VTE diagnosis, and 212 patients did not have metastasis or recurrence. In comparison between the two groups, patients with metastasis or recurrence were a strong predictor of poor prognosis (P<0.001, HR: 0.268, 95%CI: 0.197–0.364).

**Table 6:** Table 6 Univariate and multivariate analysis of risk factors for overall survival in CAT patients

	Univariate analysis			Multivariate analysis		
	HR	P value	95%CI	HR	P value	95%CI
DVT Symptoms						
Symptomatic DVT (n=100)	0.650	P=0.007	0.477–0.887	0.891	P=0.514	0.629–1.261
Asymptomatic DVT (n=222)						
Location of DVT						
Proximal (n=195)	0.596	P=0.001	0.434–0.818	0.739	P=0.089	0.521–1.047
Distal (n=127)						
D-dimer level						
≧6 µg/mL (n=211)	0.493	P<0.001	0.345–0.704	0.634	P=0.016	0.438–0.917
<6 µg/mL (n=98)						
Metastasis/Recurrence						
+ (n=110)	0.268	P<0.001	0.197–0.364	0.289	P<0.001	0.210–0.398
− (n=212)						

VTE: venous thromboembolism; DVT: deep vein thrombosis; HR: hazard ratio

The results of multivariate proportional hazards analyses using the forced entry method are shown in **Table 6**. The significance of the variables was significant for metastatic/recurrent cases (P<0.001) and a D-dimer level 6 µg/mL or higher (P=0.016). The HR was high for metastasis/recurrence, followed by a D-dimer level 6 µg/mL or higher. Metastasis/recurrence is obviously a strong poor prognostic factor, but a D-dimer level 6 µg/mL or higher was also found to be an independent and significantly poor prognostic factor.

## Discussion

Since Trousseau’s report,^4)^ it is known that the frequency of thromboembolism is higher in patients with malignancy. The annual incidence of VTE has been estimated to be 0.5% in patients with cancer, compared to 0.1% in the general population.^5)^ Twenty percent of patients with VTE have active cancer.^6,7)^ Thrombosis is the second leading cause of death in malignant tumors after progression of the primary disease,^1)^ and the prevention and treatment of VTE are important. The process of thrombus formation in CAT is different from that in nonCAT. In CAT, tissue factor (TF) is abnormally produced and converted to TF/FIIa, which activates the extrinsic coagulation pathway.^8)^ As a result, a large amount of thrombin is produced, causing hypercoagulability. In addition to the production of TF, cancer cells produce inflammatory cytokines, such as interleukins, which cause endothelial damage, and tumor-associated cysteine proteases directly activate coagulation factor Xa.^8)^ During the course of treatment, patients with malignant tumors often have multiple risk factors for thrombus formation, such as long-term indwelling catheters, chemotherapy, surgical treatment, and prolonged bed rest owing to tumor drainage and pain.^2)^ The difference between men and women varies, according to reports.^9,10)^ In terms of left–right differences, DVT is more common in the left lower extremity because of arterial compression.^9)^ In our study, DVT of the left lower extremity was more common in patients with nonCAT, whereas DVT of the bilateral lower extremity was more common in patients with CAT, with little difference between the left and right sides, which seems to be a characteristic trend in patients with CAT.

The risk of VTE varies with the type of cancer. Hematological cancer, lung cancer, pancreatic cancer, stomach cancer, ovarian cancer, and brain cancer are reported to have a high risk of thrombosis,^11,12)^ whereas prostate cancer and breast cancer have a relatively low risk of thrombosis.^13)^ In our study, patients with colorectal cancer, lung cancer, and prostate cancer had a higher incidence of VTE. This may be related to the large number of patients with prostate cancer, despite the low relative risk.^14)^ Patients with cancer are often subjected to schedule systemic imaging. Therefore, asymptomatic DVT and PTE were more frequent than symptomatic DVT and PTE because of incidental detection by imaging or screening.

Basically, anticoagulation for CAT also follows the general principles of VTE treatment, but the efficacy and safety of anticoagulation differ between patients with CAT and nonCAT. In the European and American guidelines for the treatment of VTE, LMWH is the first choice for patients with cancer. In Japan, however, LMWH cannot be used for VTE treatment, and warfarin and DOAC are used for this purpose. The administration of warfarin to patients with cancer is strongly influenced by diet, and drug interactions with fluoropyrimidine anticancer agents make it difficult to adjust the dose in many cases. Therefore, DOACs, which directly inhibit factor Xa, are attracting attention as a treatment for CAT.^15)^ DOAC is also thought to be effective in cases of thrombus formation due to TF, which is characteristic of patients with cancer.^16)^

There are still no clear criteria for how long anticoagulation should be continued for VTE in patients with cancer. The risk of VTE recurrence in patients with active cancer is about three times higher than that in patients without cancer.^17,18)^ VTE in patients with cancer should be treated for as long as possible, and anticoagulation for at least 6 months is recommended.^19)^ However, anticoagulation therapy for VTE associated with cancer treatment is known to have a higher risk of bleeding than that in patients without cancer.^18)^ In this study, many patients did not receive anticoagulation in CAT because of the risk of bleeding, prognosis, and quality of life, and many patients received low-dose anticoagulation rather than therapeutic doses of anticoagulation. In long-term treatment, it is important to evaluate the bleeding and thrombotic risks in each patient, rather than continuing standardized anticoagulant therapy. Edoxaban in the Hokusai VTE Cancer study and oral rivaroxaban in the SELECT-D study showed a lower recurrence rate of VTE but a higher incidence of serious bleeding compared with subcutaneous LMWH.^20,21)^ Apixaban in the CARAVVAGIO trial showed efficacy similar to LMWH subcutaneous injection in preventing VTE without increasing the risk of bleeding.^22)^ The efficacy and safety of low-dose anticoagulation for CAT and the risk of bleeding need to be investigated.

Most patients with malignant tumors complicated by VTE die within 5 years and have a poor prognosis.^23,24)^ In this study, we investigated the risk factors for death in relation to overall survival in 322 patients with CAT, including thrombosis factors and metastasis/recurrence. Metastasis and recurrence were obviously poor prognostic factors, but symptomatic DVT, proximal thrombus, and a D-dimer level 6 µg/mL or higher were all significant predictors of poor prognosis in univariate analyses. In multivariate analyses, a D-dimer level 6 µg/mL or higher was an independent and significant predictor of poor prognosis, as was metastasis and recurrence. In patients with DVT with malignant tumors, it has been reported that D-dimer levels are elevated in patients with more advanced tumors,^25)^ and several hemostatic biomarkers have been reported to be associated with decreased survival.^26)^ As the stage of malignancy progresses, TF and cancer procoagulant increase, and the coagulation system becomes more activated, whereas the activation of the coagulation system enhances cancer invasion and metastasis, a “positive feedback” relationship.^27)^ In patients with malignant tumors complicated by VTE, factors of thrombosis such as elevated D-dimer levels, proximal thrombus, and symptomatic DVT may be prognostic factors of malignancy as well as tumor markers. Therefore, the prevention and management of VTE in patients with malignant tumors may be important to improve the prognosis and quality of life of patients as well as the treatment of malignant tumors.

## Conclusion

Patients with CAT have a different background from patients with nonCAT. Various factors are predictive of prognosis in patients with CAT, and the prevention and treatment of VTE are as important as the treatment of the primary disease in the treatment of malignancy. Further studies are needed on the prevention and treatment in CAT.

## Appendix

Conference presentation: The 39th Annual Meeting of the Japanese Society of Phlebology, July 4–5, 2019, WINC AICHI, Japan

This study was approved by the ethical committee of the Hamamatsu University School of Medicine (17-129).

## Notes

This is secondary publication from Jpn J Phlebol 2020; 31(3): 153–159.
